# Characterization and Modeling of Interfacial Photogating Effect in Graphene Field-Effect Transistor Photodetectors on Silicon

**DOI:** 10.1021/acsaelm.4c02268

**Published:** 2025-01-22

**Authors:** Leslie Howe, Kalani H. Ellepola, Nusrat Jahan, Brady Talbert, James Li, Michael P. Cooney, Nguyen Q. Vinh

**Affiliations:** † Department of Physics and Center for Soft Matter and Biological Physics, Virginia Tech, Blacksburg, Virginia 24061, United States; ‡ NASA Langley Research Center, Hampton, Virginia 23681, United States

**Keywords:** photogating effect, graphene, field-effect transistors, doped silicon, nanostructures

## Abstract

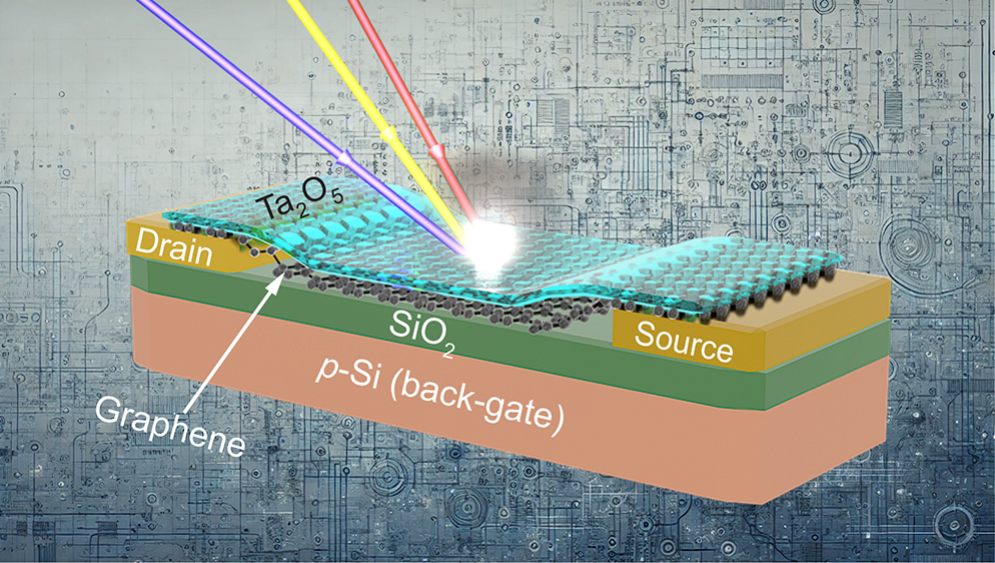

Infrared photodetection of silicon is prevented by the bandgap energy at wavelengths longer than approximately 1100 nm (∼1.12 eV) at room temperature, while silicon is the most used in modern electronics. Of particular interest is the performance of silicon for photodetectors in the infrared region beyond the silicon bandgap. Here, we demonstrate graphene field-effect transistor photodetectors on silicon with high photoconductive gain and photodetection capability extending to the infrared region. These devices have a photoresponsivity of >10^6^ A/W for excitation above the silicon bandgap energy and yield a value of 35 A/W for infrared detection at a wavelength of 1530 nm. The high photosensitivity of the devices originates from the photogating effect in the nanostructures and a long Urbach tail extending into the infrared region. A model to explain the mechanism of the photoconductive gain is proposed, which shows that the gain results from modulation of the surface charge region under illumination. The gain strongly depends on the excitation power, due to carrier capture processes occurring over the barriers associated with the surface charge region, in agreement with the experimental data. This model properly explains the photoresponse behavior of graphene field-effect transistors on silicon.

## Introduction

Photodetectors fabricated from low-dimensional substances, including two-dimensional (2D) materials, nanowires, and quantum dots, have demonstrated high potential for applications based on observations of extremely high photoresponsivity.^1−3^ In these low-dimensional structures, the photogating effect strongly contributes to the conversion of light into electrical signals.^4−9^ For example, in a field-effect transistor structure, the photogating effect modulates the conductance of the device channel through a photoinduced gate voltage.^3,10,11^ In this situation, photogenerated carriers (electrons and holes) in the active area of the device are separated owing to the interfacial potential induced by interfaces in the low-dimensional structures.^3−7,12^ If one type of photogenerated carrier is captured at the interfacial potential, it produces an extra gate voltage that regulates the conductance of the active channel. Since the lifetime of carriers in trapped states is prolonged, and the transit time of induced carriers is short, a high photoresponsivity in these low-dimensional structures can be obtained. Typically, the photoconductive gain, *G* = τ_trapped_/τ_transit_ (τ_trapped_ is the carrier lifetime in trapped states, and τ_transit_ is the transit time of the opposite charge carrier in the channel), which is commonly used to evaluate the photoconductive gain in low-dimensional structures of photodetectors.^3^

Graphene in the field-effect transistor structure (graphene/SiO_2_/p-Si) for photodetection inducing a strong photogating effect provides an ultrahigh photoconductive gain for the device. Specifically, doped silicon wafers (p- or n-type material) at the back-gate of the device are utilized as efficient absorbers, instead of only supporting parts of the devices.^12−16^ The difference in the work functions of graphene, doped-Si, and SiO_2_ induces a potential well at the SiO_2_/p-Si interface to trap photogenerated carriers (negative or positive charges), thus generating an extra voltage to regulate the carrier concentration in the graphene channel of the device through the coupling capacitor. A graphene layer with a high carrier mobility can sense changes in the surface charge region at the SiO_2_/p-Si interface, including the width of the surface charge region and surface charge density, generating an additional photogating voltage. These factors result in a high photoconductive gain in the graphene field-effect transistors (GFETs).^12−16^

To elucidate the photogating effect of GFETs on the photodetection performance, we fabricated, characterized, and proposed a model for the photoconductive mechanism of GFET photodetectors on p-type Si substrates (Si:B). GFET photodetectors on p-doped Si substrates present a strong photoresponse to the band-to-band excitation of silicon, with a photoresponsivity higher than 10^6^ A/W and obtain a high performance under infrared (IR) illumination (∼35 A/W under 1530 nm excitation). The sub-bandgap photoresponse is related to the absorption tail, characterized by an Urbach energy of 29.9 meV for a doping concentration of 3 × 10^15^ cm^–3^. We propose a photoconductive gain model that considers the effect of modulation of the surface charge region and surface charge density under illumination. This illustrates that the photogating effect significantly contributes to the photoconductive gain of the GFETs. The proposed model provides quantitative agreement with the experimental results, including the power and temperature dependences of the photoconductive gain.

## Device Fabrication and Characterization

**Figure 1:**
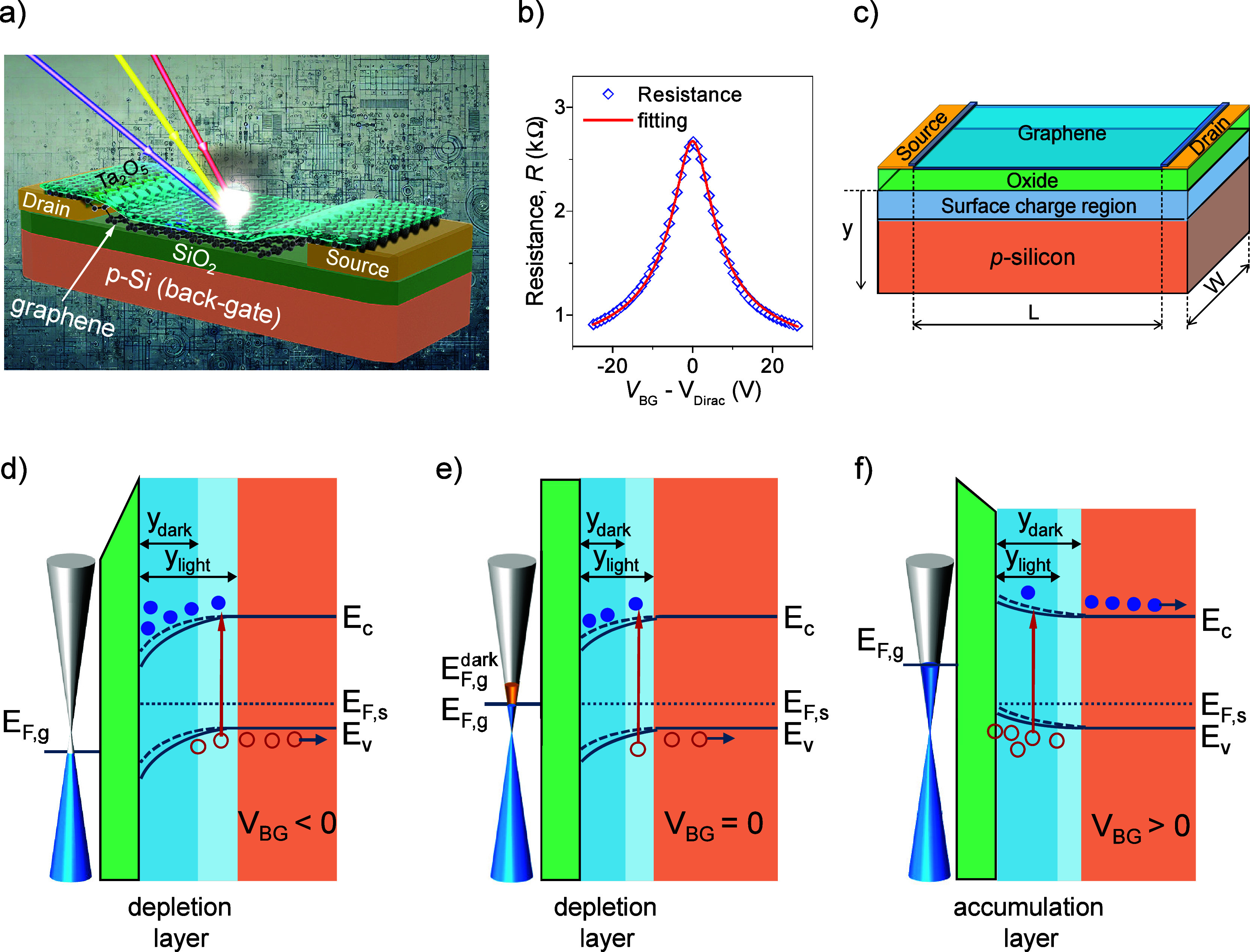
Schematic illustration of GFET photodetectors based on photogating effect on p-type Si substrates. (a) Schematic structure of a GFET photodetector, including Al_2_O_3_/graphene/SiO_2_/p-Si structure, source and drain contacts. (b) Resistance–voltage transfer curve of a GFET device (*L* = 10 and *W* = 20 μm) under dark conditions and *V*_DS_ = 0.2 V at room temperature for determining the mobility of carriers in the graphene channel. (c) Schematic diagram illustrating parameters used in the modeling to understand the performance of the devices, including experimental dimensions, the graphene/insulator/semiconductor for the photogating effect and the surface charge region in the p-Si back-gate. (d–f) Energy band diagrams of heterostructures of graphene/SiO_2_/p-Si indicating the band bending, thickness of the surface charge region with and without illumination, and the shift of the graphene Fermi level at different back-gate voltage conditions, forming depletion and accumulation layers. Open red dots are holes, and blue dots are electrons.

GFETs were fabricated on p-type boron-doped silicon (Si:B) wafers (1–10 Ω cm) with a doping concentration of ∼3 × 10^15^ cm^–3^ which is well below the critical concentration of the Mott transition forming metallic state.^17^ Initially, a 290 nm silicon oxide layer was thermally grown on these silicon wafers at 1,100 °C. Ohmic contacts (5 nm Cr and 80 nm Au), including the back-gate, drain, and source, were formed using electron-beam (e-beam) evaporation, photolithography, and metal liftoff steps. Next, a single layer of chemical vapor deposition graphene was transferred onto the SiO_2_/p-Si substrate.^14,18^ Photolithography as well as oxygen-plasma dry etching were applied to establish the graphene channel, including the distance between the source and drain contacts, *L*, of 10 μm, and width, *W*, of 20 μm. To protect the surface of graphene, a 3 nm nucleation layer of Ta_2_O_5_ was deposited by e-beam evaporation method on top of the graphene channel, afterward, a 25 nm Ta_2_O_5_ film was deposited by atomic layer deposition (ALD) method on the devices (Figure 1). Finally, the oxygen-plasma dry etching was used to reopen electrical contacts, including back-gate, drain and source terminals.

The photoresponse characteristics of GFET devices with and without light radiation were characterized using source-meter units at room temperature.^14^ A source-meter unit (Keithley 2450) was applied to fix the voltage between the drain and source contacts, *V*_DS_, of 0.2 V as well as to detect the drain current, *I*_D_, while the second source-meter unit was employed to sweep the bias voltage on the back-gate, *V*_BG_, between −25 to 25 V. A lock-in amplifier was used to collect the photocurrent under on/off and low illumination conditions. For the optical characterization, a number of lasers with wavelengths of 532 and 1530 nm, and a broad-band visible to near IR light emitter from an Edmund tungsten lamp were employed to characterize the photoresponse of the GFETs. Narrow bandpass spectral filters with a fwhm of 10 nm were used to select the desired wavelengths. Continuous-wave laser beams were turned off and on using an acousto-optic modulator or mechanical choppers. Details of the device fabrication processes as well as electrical/optical characterizations of the GFET photodetectors are provided in the Supporting Information.

The mobility of carriers in the graphene channel is a critical parameter for the optical performance of GFET photodetectors.^13,15,19,20^ To achieve high carrier mobility in graphene, a high-κ dielectric film (Ta_2_O_5_) with a dielectric constant (ε ∼ 25–50), was deposited on the graphene channel by the ALD method.^12,21−24^ The screening effect of the Ta_2_O_5_ layer can minimize the degradation of carrier mobility in graphene,^23,25,26^ leading to an improvement in the optical performance of these photodetectors.^23,27,28^ The ALD Ta_2_O_5_ capping layer does not absorb photons with energy from the ultraviolet (UV) to the IR region; thus, no additional optical signal originates from this layer.^29,30^ The carrier mobility, μ, can be obtained from the transfer curve (or drain current vs. back-gate voltage characteristic) of the GFET by fitting the resistance of the graphene channel, *R*, under dark conditions to the expression,^31−33^
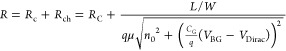
1where *q* is the elementary charge, *R*_ch_ is the resistance of the graphene channel, *R*_c_ is the total contact resistance,  ∼ 11.3 nF/cm^2^ is the capacitance of the gate per unit area, ε_0_ is the vacuum permittivity, ε ∼ 3.7 is the dielectric constant of SiO_2_,^34^*d* = 290 nm is the SiO_2_ thickness, *n*_0_ is the concentration of carriers generated by charged impurities at the graphene/SiO_2_ interface, and *n*_g_ =  is the carrier concentration created by a bias applied on the back-gate away from the Dirac point voltage, *V*_Dirac_, or the charge neutral point (CNP) voltage. The best fit (red line, Figure 1b) to the transfer curve revealed a carrier mobility of ∼5,080 ± 250 cm^2^ V^–1^s^–1^ in the graphene channel.

**Figure 2:**
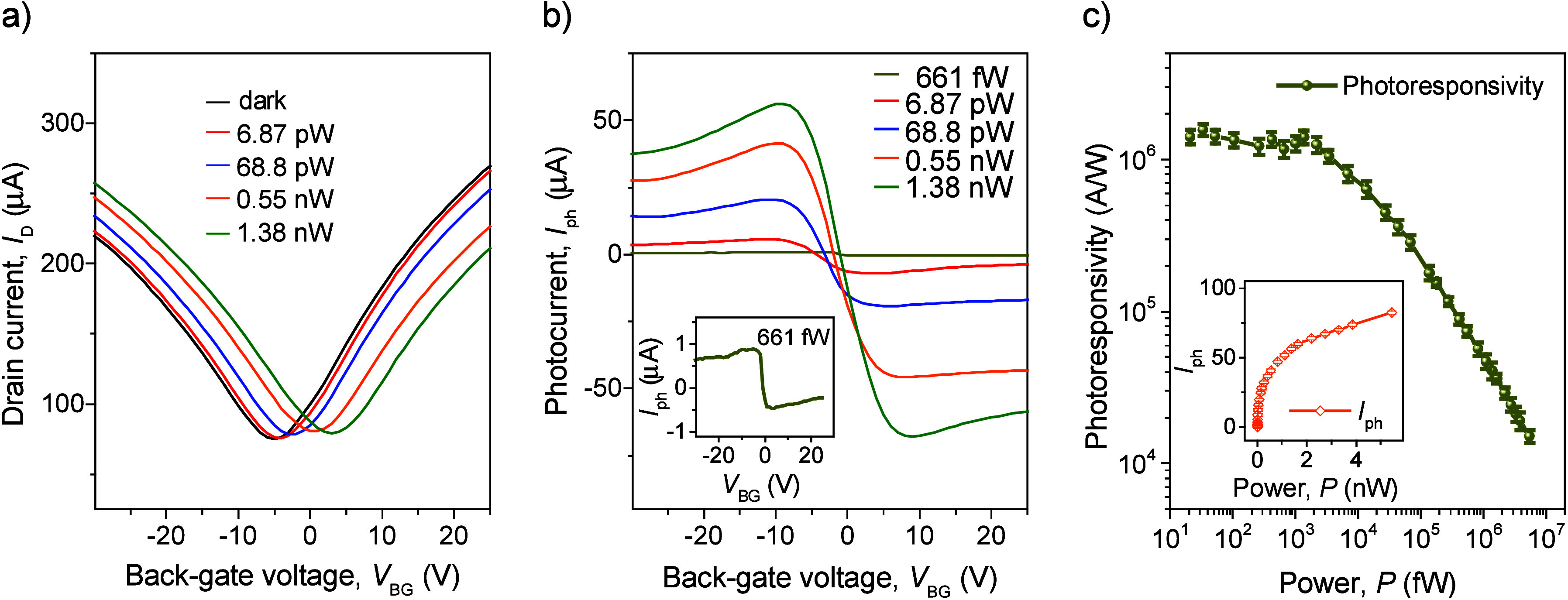
Photoresponse characterization of a GFET photodetector at room temperature, under*V*_DS_ = 0.2 V and λ = 532 nm. (a) Current–voltage transfer curves of the GFET photodetector with and without illumination, showing a shifting of the CNP toward the positive value of the back-gate voltage. (b) Photocurrent in response to the back-gate voltage under different excitation powers. Inset shows the photocurrent under an excitation power of 0.661 pW. (c) Photoresponsivity of the GFET photodetector in response to the excitation power, showing an approximately constant value at low excitation intensity and lower values at high power. Inset shows the photocurrent in response to the excitation power.

The effect of converting light into electric current of the GFET is shown in Figure 2. Specifically, transfer curves were obtained with and without illumination at 532 nm under *V*_DS_ = 0.2 V. The intensity of the light varied from femto- to nanowatts (Figure 2a). With increasing illumination power, a shift in the transfer curves, corresponding to the moving of the CNP voltage, is observed toward positive values of the back-gate voltage, revealing the p-doped characteristic property of the graphene channel. The photocurrent is estimated as *I*_ph_ = *I*_light_ – *I*_dark_, where *I*_dark_ and *I*_light_ are the electric currents under dark and illumination conditions, respectively. The photocurrent in response to the back-gate voltage is shown in Figure 2b. The maximum photocurrent is obtained at *V*_BG_ ∼ −9 V under different excitation intensities, as a result of the maximum efficiency of the bending of energy bands at the SiO_2_/p-Si interface for the photogating effect. The photocurrent at *V*_BG_ = −9 V under different excitation intensities is presented in Figure 2c, inset. The detector is highly sensitive to light. Under a weak excitation power from 20 fW to 10 pW, the photocurrent grows linearly and increases slowly under high illumination intensity. A photocurrent of ∼0.775 μA is obtained at an excitation intensity ∼0.661 pW.

For imaging and remote sensing applications, a wide range of photodetection is an important property of photodetectors. To estimate the detectivity of the devices, their photoresponsivity is determined as, *R*_ph_ = *I*_ph_/*P*, where *P* is the power of the light source. Figure 2c shows photoresponsivity as a function of the excitation power. Under low illumination power, the photocurrent increases linearly with power, indicating a constant photoresponsivity. A high photoresponsivity of ∼1.2 × 10^6^ A/W is obtained across almost 3 orders of magnitude of excitation intensity on the femto- to picowatt level at λ = 532 nm, allowing for weak detection of optical signals. In this situation, free holes and electrons produced from the photoabsorption process are well separated; thus, a higher excitation intensity induces a higher photocurrent. However, when the excitation illumination exceeds a certain value, a large number of electron–hole pairs are created. Several recombination channels of holes and electrons occur, including radiative, nonradiative, and Auger recombination processes;^12,14,35^ thus, these holes and electrons do not support the photocurrent in the graphene channel. Moreover, the number of photons exceeded the absorption limit in the active region of the SiO_2_/p-Si interface (∼10 nm), thereby reducing the photoresponsivity. At a high illumination intensity, the rate of increase of the photocurrent in response to the increasing excitation power slows down, and the photoresponsivity decreases.

**Figure 3:**
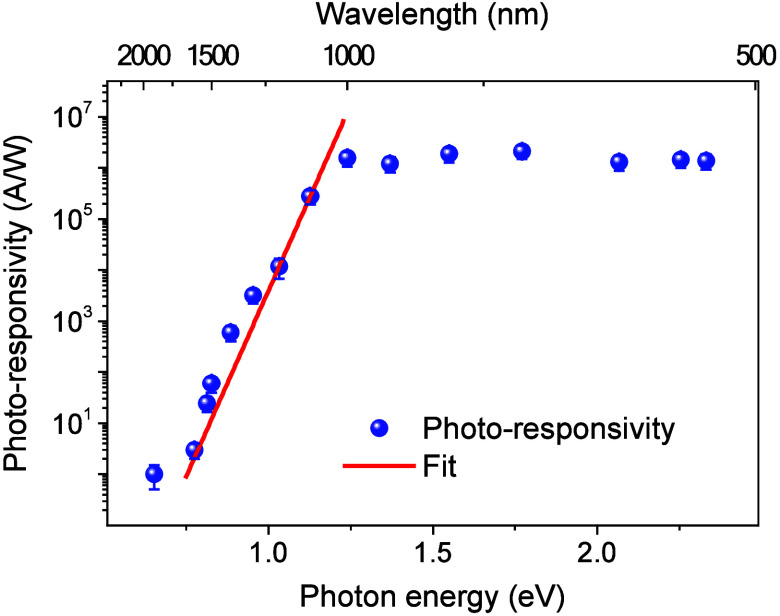
Wavelength dependence of the GFET photodetector. Photoresponsivity of the GFET on p-Si substrate increases significantly as photon energy is higher than the silicon bandgap under *V*_DS_ = 0.2 V at room temperature.

GFET photodetectors on p-doped Si substrates convert optical signals into electric currents over a large spectral range with high sensitivity in the UV to near IR region. The photoresponsivity of the devices was characterized at room temperature, normalized by optical excitation, and plotted on a logarithmic scale (Figure 3). The photodetectors obtain a high photoresponsivity under the overbandgap excitation of silicon (>10^6^ A/W) and achieve a high performance under IR illumination, *V*_DS_ = 0.2 V, and *V*_BG_ = −9 V. The onset of photocurrent at 0.75 eV (∼1650 nm) was identified for the photodetection of these devices.

The devices detect photons in the near IR region with photon energy well below the bandgap energy of silicon (∼1.12 eV at room temperature). Specifically, a high photoresponsivity of ∼35 (A/W) under 1530 nm (∼0.81 eV) excitation is obtained (Figure 3). The photoresponse of GFETs in the near IR region originates from the high photoconductive gain and below-bandgap absorption tail (or Urbach tail) of silicon. The absorption tail is defined as the density of states that extend from the energy bands into the bandgap of the material as an exponential absorption edge. The tail changes from two to 4 orders of magnitude and appears in disordered materials, including strains, defects, and doping elements in crystalline materials. Additionally, silicon is an indirect bandgap semiconductor, which typically has a long absorption tail into the long-wavelength region, enhancing the photoresponse of the devices in the near IR region. Most significantly, the high doping concentration of boron in silicon further increases the absorption of silicon into the IR region.^36,37^ The below-bandgap photocurrent related to the sub-bandgap absorption, which exponentially depends on the photon energy and follows the empirical Urbach equation,^38,39^ is expressed as
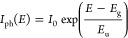
2where *I*_0_ is the photocurrent induced from photons with energy equal to the bandgap energy, *E*_g_, of silicon and *E*_u_ represents the Urbach energy associated with the density of states in the silicon bandgap. We obtain the best fit of the spectral response for *E*_g_ = 1.12 eV and *E*_u_ = 29.9 ± 3.5 meV (Figure 3). The Urbach energy from the spectral response is in good agreement with absorption spectra of doped silicon materials with an energy value of ∼35 meV.^37,39−41^ Note that the IR photoresponse of these devices obtains the highest signal under a moderate doping concentration of the order of ∼10^15^ cm^–3^. At high doping concentrations, the photoresponsivity was significantly reduced.^14^ In this situation, nonradiative recombination processes, including Auger recombination, defects, and trap-assisted pathways for electron–hole pairs, become dominant in highly doped silicon materials, strongly reducing the photoresponsivity. In addition, highly doped materials produce a large number of strains and defects at the SiO_2_/p-Si interface, resulting in a deficiency of the photogating effect for photodetection.

## Modeling of the Photogating Effect in GFETs

The operation of GFET photodetectors on a p-doped Si substrate under band-to-band illumination, together with Urbach tail photon excitation is based on the interfacial photogating effect (Figure 1).^3,5,7,12,13,42^ Specifically, the work function of graphene and the p-Si substrate are about 5.07 and 4.56 eV, respectively.^43−46^ The mismatch of energy in the work function leads to downward band bending for the p-Si substrate at the SiO_2_/p-Si interface. The downward bending of the energy bands forms a surface potential in the p-Si region that captures electrons near the interface.^14^ Under excitation, electrons (blue dots) are excited to the conduction band and trapped at the SiO_2_/p-Si interface, whereas opposite charges (holes, open circles) are repelled toward the metal contact of the back-gate. The trapping of photogenerated electrons in the potential well generates an extra negative voltage at the SiO_2_/p-Si interface, inducing an increase in the hole current in the graphene channel. Thus, the transfer curve of the GFET photodetectors shows a shift to the positive back-gate voltage under illumination.

The potential of the photogating effect strongly depends on the charge density and energy bands of the p-Si semiconductor at the interface under illumination (solid lines) and dark (dash lines) conditions (Figure 1). The capacitive coupling effect of graphene as a conductive layer through the dielectric layer (SiO_2_) produces a high density of interfacial states for carriers in the p-Si semiconductor. From the viewpoint of semiconductor for p-Si substrates, the configuration is analogous to metal–insulator-semiconductor (MIS) or metal-oxide-semiconductor field-effect transistor (MOSFET) structures. No current flows from the graphene channel to the p-Si back-gate, resulting in the flattening of Fermi levels. When the GFET structure is biased, due to the low concentration of carriers in the p-doped silicon, the surface charge region is built up at the silicon interface. In this region, the charge carrier concentration is depleted compared to that in the bulk, forming an electric field. Thus, the energy band edges of the p-Si were continuously bent.

Energy band bending strongly depends on the voltage applied to the back-gate of GFETs, generating a photocurrent in the graphene channel. When a negative voltage is applied to the p-Si back-gate and the bias between the drain and source of the graphene channel is kept at a low level of 0.2 V compared to the back-gate voltage, the silicon energy bands bend downward at the SiO_2_/p-Si interface. Hole carriers are depleted, forming a depletion layer (Figure 1d). Under band-to-band excitation, electron–hole pairs are generated in the p-Si substrate and separated by the electric field. Holes are moved away from the interface, and electrons are trapped at the p-Si interface, broadening the depletion region, thus increasing the width of the space charge region (*y*_light_). To neutralize the electrostatic charges, an increase in the drain current has been observed in the graphene channel. At the *V*_BG_ = 0 V, the energy bands are slightly bent down at the silicon interface due to the mismatch of the Fermi energy between graphene and silicon (Figure 1e). Under illumination, a similar process has been observed. When a positive voltage is applied to the back-gate, the energy bands near the silicon interface bend upward, causing the accumulation of holes (majority carriers) near the silicon surface or forming an accumulation layer (Figure 1f). Under illumination, electrons generated from the absorption process are attracted to the positive electrode. Holes accumulate near the SiO_2_/p-Si interface and are captured at surface states originating from dangling bonds on the native oxide interface,^41,47,48^ lowering the surface potential and decreasing the drain current, *I*_D_. Thus, the graphene channel associated with the drain current can sense potential changes in the surface charge region during sweeping of the back-gate voltage. The effective electric field originating from the trapped carriers induces a strong photogating effect, appearing as a horizontal shift of the *I*–*V* transfer curve of the GFETs under illumination. The circulation of the charge carriers under the drain-source bias, *V*_DS_, in the graphene channel within the lifetime of the trapped charges in the SiO_2_/p-Si interface contributes to a higher photoconductive gain of the devices.

The number of charges in the surface charge region (surface charge density, *Q*_SC_) at the SiO_2_/p-Si interface is equal to the number of charges in the graphene channel (*Q*_Gr_), balancing the charges in the area. The time evolution of the width of the surface charge region can be investigated, as in the case of a MOSFET on a p-type semiconductor, with hole majority carriers in the material. By solving the one-dimensional Poisson equation, the profile of potential, *V*, under dark conditions can be identified in the direction perpendicular to the interface (*y*-axis, Figure 1c), ∂^2^*V*/∂*y*^2^ = −*qN*/(εε_0_), where *q* is the electron charge and *N* is the generalized doping distribution with a negative sign for donors.^49−51^ The depletion width, *y*_dark_, of the surface charge region in the dark condition is estimated from the doping concentration and the surface band-bending potential, Δ*V*_dark_ = *V*_bi_ + *V*_effBG_, which is related to,^7,42,50,52^
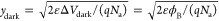
3where *V*_bi_ is the height of the surface potential, *V*_effBG_ is the effective back-gate bias at the interface, ϕ_B_ is the barrier height, and *N*_a_ is the acceptor doping concentration of the p-Si substrate. For simplicity, we assume that the band-bending potential is equal to the potential barrier, ϕ_B_. The surface charge density, *Q*_sc_^dark^, as a result of trapped or accumulated electrons, is given by,
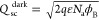
4

The graphene channel can sense the surface potential of the SiO_2_/p-Si interface, including the depletion width and surface charge density (Figure 1). Under a bias voltage between the drain and source, *V*_DS_, the drain current under dark conditions, *I*_dark_, in the graphene channel of the GFET is related to the cross-section, *S*, of the surface charge region, including the width (or depth) of the surface charge region, *y*_dark_, and the width of the graphene channel, *W*, (Figure 1b), given by Ohm’s law in the form
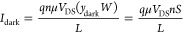
5where μ and *n* are the carrier mobility and free-electron density in graphene, respectively, *L* is the length of the graphene channel. The Fermi level of graphene can be continuously modulated by applying an electric potential to the back-gate.

Under illumination, electron–hole pairs are generated in the p-Si back-gate and separated by the electric field in the surface charge region. Electrons are trapped at the SiO_2_/p-Si interface when a negative voltage is applied to the back-gate, thus varying both the width of the surface charge region (*y*_light_) and surface charge density (*Q*_sc_). The absorption of light generates an extra voltage or photogating voltage, *V*_ph_, at the interface. The width of the surface charge region increases from *y*_dark_ to *y*_light_, which can be expressed as
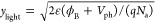
6and the surface charge density, *Q*_sc_^light^, under excitation is given by
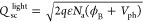
7

The trapping of photogenerated carriers in the potential well generates an extra negative voltage at the SiO_2_/p-Si interface, inducing more holes in the graphene channel through the coupling capacitor of the insulating SiO_2_ layer. A new equilibrium state of the interface is constructed. Under illumination, an additional photocurrent is generated, given by
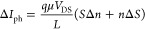
8where Δ*n* is the variation in the carrier density in the graphene channel, and Δ*S* is the change in the cross-section of the surface charge region at the interface in the semiconductor under illumination (Figure 1). The variation in the concentration of photogenerated carriers in the graphene channel, which is proportional to the photon absorption of graphene, *S*Δ*n*, is very low. Thus, the additional photocurrent strongly depends on the variation in the cross-section, Δ*S* = *W*(*y*_light_ – *y*_dark_), of the surface charge region, estimated from the device configuration
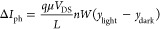
9

The surface potential at the interface strongly depends on illumination power and temperature. Under band-to-band excitation and low-power illumination, photogenerated electron–hole pairs are separated by the potential of the surface charge region within their diffusion length. Under high-power illumination, the photogenerated electron–hole pairs are closer than the diffusion length. Thus, an efficiency factor, η, is considered in the quantum efficiency. An approximation of the illumination power can be estimated in the form of *P* = *ηP*_0_ exp(−*αz*), where α is the effective absorption coefficient of the semiconductor material (assuming that the reflection is neglected) and *P*_0_ is the incident power. We ignore the tunneling effects of carriers in the first approximation. By evaluating the variation of the width of the surface charge region, the photogating voltage of the GFET in response to the excitation intensity can be determined. In the steady state, the thermionic current released from the interface, *I*_released_ = A*T^2^ exp(−*qϕ*_B_/(*k*_B_*T*)) = A* *T*^2^ exp(−*ϕ*_B_/*V*_T_), balances the thermionic current of carriers trapped at the interface, *I*_trapped_(*t*) = A*T^2^ exp(−*qϕ*_B_/(*k*_B_*T*)) exp(*qV*_ph_/(*fk*_B_*T*)) = A*T^2^ exp(−ϕ_B_/*V*_*T*_) exp(*V*_ph_/(*fV*_T_)), where *k*_B_ is the Boltzmann constant, *T* is the temperature in Kevin (K), A* is the effective Richardson constant,^53^*V*_T_ = *k*_B_*T*/*q*, and *f* is the ideality factor.^42,50^ Thus, the dynamic equation of accumulated charges can be defined as
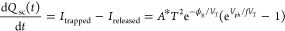
10At equilibrium under illumination, the photovoltage will appear when *I*_trapped_ – *I*_released_ = *qP*/(*hν*); thus, the well-known Schottky diode photovoltage equation for the structure is in the form
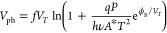
11

The gain of the photoconductive current of the GFET photodetector can be estimated as,
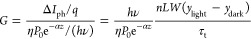
12where *hν* is the photon energy, ν is the frequency of the photon, *h* is Planck’s constant, *τ*_t_ = *L*/*s* = *L*^2^/(*μV*_DS_) is the transit time of carriers in the graphene channel, and *s* is the carrier velocity. Thus, we obtained the following expression for the photoconductivity gain,
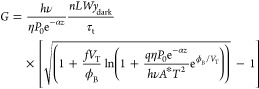
13A similar format for the photoconductive gain can be obtained under the accumulation conditions (Figure 1f). In this situation, the width of the surface charge region reduces from *y*_dark_ to *y*_light_, and the photoconductive gain is given as
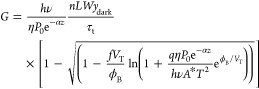
14

**Figure 4:**
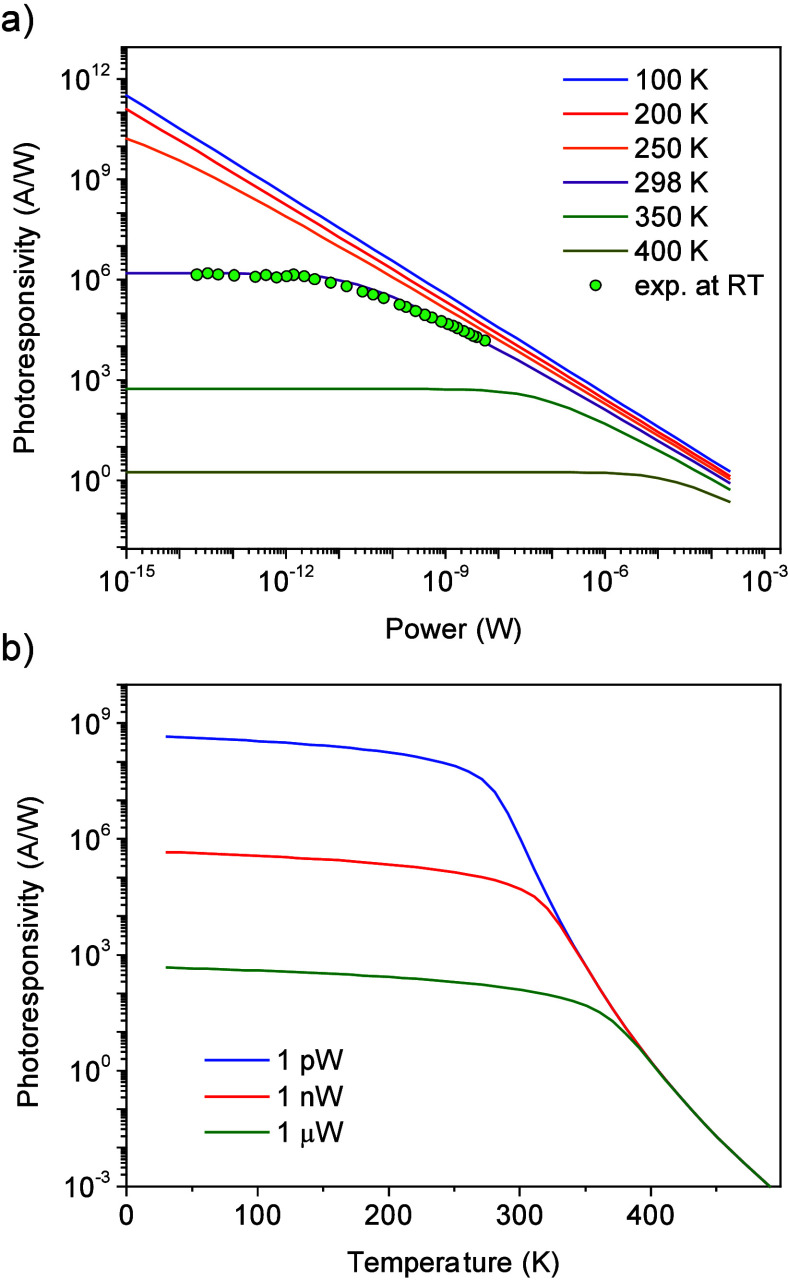
Computational simulations of the photoconductive gain based on the photogating effect. Photoconductivity strongly depends on the (a) excitation power and (b) temperature of the device. The simulations were performed under *V*_DS_ = 0.2 V, *L* = 10, and *W* = 10 μm.

The above model can provide an estimation of the desired photoconductive gain in the graphene channel for different optical excitation powers, temperatures and device structure parameters. The simulations of the photoconductive gain as a function of the excitation power are provided in Figure 4 for different temperatures. In this simulation, the following parameters were used: length and width of the graphene channel of 10 and 20 μm, respectively, doping of 3 × 10^15^ cm^–3^, effective Richardson constant of 150 A cm^–2^ K^–2^,^50,53^ transit time of carriers in the graphene channel of 0.47 ns corresponding to the mobility of carriers of ∼5,080 cm^2^ V^–1^s^–1^ in the graphene channel, excitation wavelength of 532 nm with an absorption coefficient of silicon of 7.85 × 10^3^ cm^–1^, efficiency factor of 0.5, and barrier height ϕ_B_ = 1.35 V. Thus, under the dark conditions, *y*_dark_ ∼ 750 nm and under the illumination conditions, *y*_light_ varies from 400 to 1000 nm, depending on the incident power, drain-source bias, back-gate voltage, absorption coefficient at the excitation wavelength and device temperature. Note that the bending of the energy bands is induced by the total surface charge, which is a combination of the total charges at the surface and the effective voltage applied to the back-gate. These values provided the best fit for the experimental data.

The photoconductive gain strongly depends on the excitation power and temperature with a logarithmic function, despite the square-root dependence of ϕ_B_ in eqs 11 and 13. At room temperature, *T* = 298 K, the flat photoconductive gain part occurs at extremely low excitation powers in the femto- to pico-watts due to the low *V*_ph_ values that evolved during illumination in the surface charge region. Under these conditions, the number of carriers swept per second by the surface charge region is low compared with the fluxes of thermionic electrons over the barrier, which is equivalent to the linear behavior of the photocurrent with excitation power (eqs 11 and 13). At a high excitation power the photoconductive gain decreases with a power law relation of the excitation powers, *G* ∝ *P*^–β^,^42^ corresponding to a straight line in the log–log plot (Figure 4a). The slope of the curves strongly depends on the temperature. At low temperatures, for example, at *T* = 100 K, the photoconductive gain in the log–log plot exhibits a linear dependence with β = −0.992, which is close to −1. The absolute value of β decreases at high temperatures, and the values of β are −0.969, −0.944, −0.911, −0.841, and −0.637 at device temperatures of 200, 250, 298, 350, and 400 K, respectively. This behavior has been reported for low-dimensional photodetectors and photovoltage field-effect transistors.^3−5,7,35,42^ To understand the temperature dependence of the photoconductive gain of the graphene channel, we plotted the photoresponsivity as a function of the temperature for a fixed excitation power (Figure 4b). Depending on the excitation power, the photoconductive gain reduces slowly with increasing temperature up to 300 K and significantly drops off at higher temperatures. Additional calculations for the photoconductive gain are provided in the Supporting Information, including the parameters of the device structure and electric voltages applied to the device.

**Figure 5:**
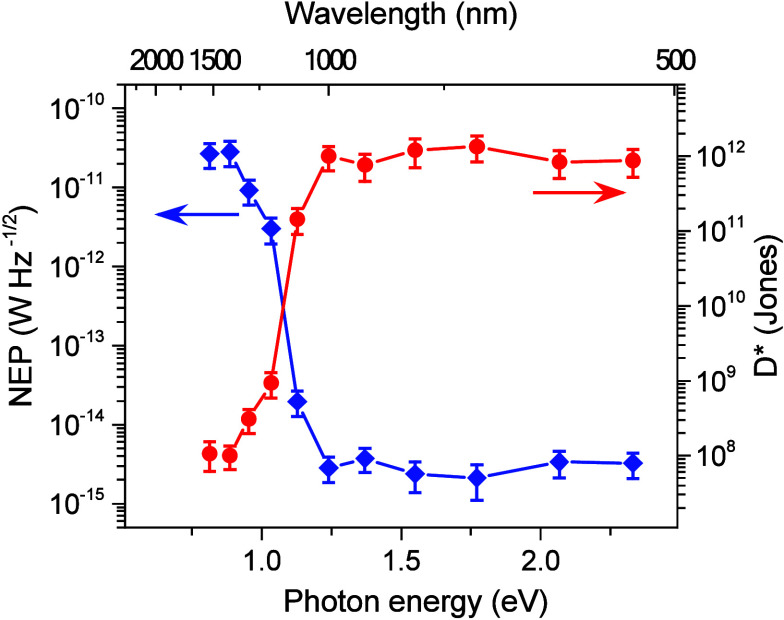
Noise equivalent power and specific detectivity of the graphene field-effect transistor photodetector as a function of photon energy and wavelength. The noise measurements were characterized under *V*_DS_ = 0.2 V.

For practical applications of the photodetectors, the noise equivalent power (NEP) which is a metric to evaluate the sensitivity of a photodetector has been characterized. The NEP is the signal power that produces a signal-to-noise ratio of unity within the standard bandwidth of 1 Hz (i.e., the root-mean-square noise level of the dark-current divided by the responsivity of the detector) given by^23,27,54^
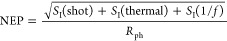
15where *S*_I_(shot), *S*_I_(thermal), and *S*_I_(1/*f*) are the shot noise, thermal and power spectral density of the 1/*f* noise, respectively, for 1 Hz bandwidth. The power spectral density of the 1/*f* noise of the photodetector has been characterized using a single-channel 100 kHz FFT spectrum analyzer (SR770).^12,18,23^ The power spectral density values of *S*_I_(shot), *S*_I_(thermal), and *S*_I_(1/*f)* noise for 1 Hz bandwidth are 3.31 × 10^–25^, 2.40 × 10^–23^, and 1.36 × 10^–17^ A^2^/Hz at *V*_DS_ = 0.2 V. We have obtained the NEP values from 2.7 × 10^–11^ (at λ = 1530 nm) to 3.2 × 10^–15^ W Hz^–1/2^ (at λ ∼ 500 nm) in the detection range from the visible to the NIR region, under *V*_DS_ = 0.2 V. From the NEP values, we have estimated the specific detectivity, D* (Figure 5).
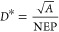
16where *A* is the area of the graphene channel. The specific detectivity, D*, is in the range from 1.0 × 10^8^ to 8.7 × 10^11^ Jones (cm Hz^1/2^/ W) in the visible to the NIR region. The significantly small NEP values indicate that the photodetectors are suitable for weak light detection.

## Conclusions

We have demonstrated an approach and proposed a comprehensive model to obtain high-performance broadband GFET photodetectors on silicon based on the photogating effect. The device demonstrates a high photoresponsivity (>10^6^ A/W) in the near IR to UV region, with photon energy higher than the silicon bandgap. Photocurrent spectroscopy reveals a high photoresponse below the silicon bandgap down to 1650 nm, originating from the high photoconductive gain based on the photogating effect and the Urbach tail accompanied by a characteristic energy of 29.9 meV. The photoresponsivity of the GFET photodetector is reduced at high excitation power. A comprehensive model derived from the variation in the surface charge region is presented to explain the large photoconductive gain of the devices, which shows that the mechanism in controlling the gain is the modulation of the conductivity of the graphene channel. The model predicts a high photoconductive gain at a low illumination power and a decrease in the gain in the high excitation regime, which is in good agreement with the experimental data. A complete study of the photoconductive gain dependencies on the power, temperature, device structure, and operating conditions has also been performed to understand the performance of the devices.

## Supporting Information Available

Details for GFET fabrication, Raman spectrum, optical and electrical setups, extra simulation results (PDF)
