# Supplementary material for: Characterization and Modeling of Interfacial Photogating Effect in Graphene Field-Effect Transistor Photodetectors on Silicon
Source: ACS Appl Electron Mater. 2025 Jan 22;7(3):1305–13. doi: 10.1021/acsaelm.4c02268 (PMC11823461; doi:10.1021/acsaelm.4c02268)
Supplement: Supplementary file 1 — el4c02268_si_001.pdf [file el4c02268_si_001.pdf]

SUPPORTING INFORMATION

Characterization and Modeling of Interfacial Photogating Effect in Graphene Field-Effect Transistor Photodetectors on Silicon

Leslie Howe,[1] Kalani H. Ellepola,[1] Nusrat Jahan,[1] Brady Talbert,[1] James Li,[1]
Michael P. Cooney,[2] and Nguyen Q. Vinh[1]*

[1] Department of Physics and Center for Soft Matter and Biological Physics, Virginia Tech, Blacksburg, VA 24061, USA

[2] NASA Langley Research Center, Hampton, Virginia 23681, USA

* Corresponding author: vinh@vt.edu; phone: 1-540-231-3158

1. Device fabrication

Graphene field-effect transistor (GFET) photodetectors have been fabricated on *p*-type boron-doped silicon (Si:B) wafers with a doping concentration of 3.0×10^{15}. To begin, we removed the native oxide layer from the surface of the silicon wafers which serve as the substrate or back-gate of the devices. After submersion in a buffered oxide etch (BOE) bath (HF based etchant), each wafer was cleaned thoroughly with deionized water in a spin-rinse-dry (SRD) machine before the fabrication of an oxide layer. An oxidation furnace was used to grow the oxide layer on the surface of the substrate. This was done by heating the samples to 1100 °C, and flowing oxygen at a flow rate of 0.8 sccm through the chamber to allow the silicon to react and form silicon dioxide. The total thickness of the oxide layer is 290 nm, with the middle part (~190 nm) being fabricated using wet oxidation, and the first and final layers (~50 nm) being grown with dry oxidation. The oxidation allowed for a high-quality interface with both the substrate and metal contacts which were deposited later on the oxide. After this step, the oxide layer was etched to be able to deposit a back-gate contact directly on the *p*-Si substrate.

To create back-gate contacts, a buffered oxide etch bath was used to etch the oxide insulating layer. First, a positive photoresist (S1813) was spun onto the wafer at a speed of 2000 rpm for 45 seconds. After a 1-minute soft bake at 100 °C, the wafer was exposed to UV light under a photomask to shape the back-gate contact and developed in an MF-319 developer for 1 minute. After the photoresist was deposited and

shaped, we submerged the wafers in a (BOE) bath for 4 minutes, until the samples were etched onto the *p*-Si material. Subsequently, the back-gate contact was deposited using physical vapor deposition (PVD-250). Here, we deposited 5 nm of chromium to ensure a strong bond to silicon and then 80 nm of gold for the metal contacts. A lift-off step was used to remove excess gold to complete the substrate fabrication.

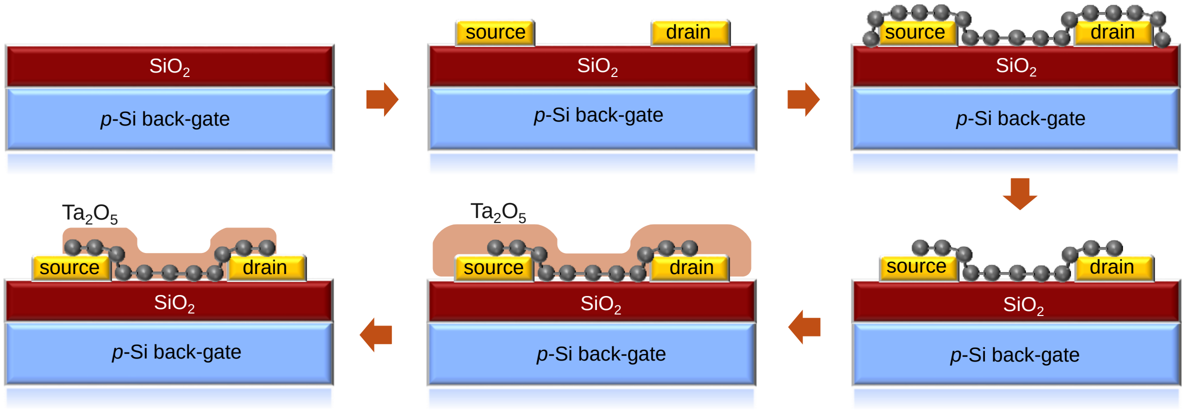

Figure S1. A schematic diagram showing fabrication steps of GFET photodetectors.

Next, metal contacts that served as the source and drain terminals for the photodetector were fabricated. The spin-coating method was applied by spinning a negative photoresist (AZ2020) on the surface of the wafer at a speed of 2000 rpm for 45 seconds, followed by a soft bake step at 110 °C for 80 seconds. Following the deposition of the photoresist, the wafer was exposed to UV light under a photomask, undergone a hard-bake step at 110 °C for 1 minute, and then developed with MIF-300 for 1 minute. Once the photoresist was patterned appropriately, PVD-250 was used again to deposit metal source and drain contacts with 5 nm chrome and 80 nm gold. After the gold-lifting-off step, the metal contacts for the source and drain of the device were completed.

A graphene layer was deposited between the source and drain contacts to serve as an electrical channel for charge carriers to flow. Graphene was procured from Graphenea and grown by chemical vapor deposition (CVD) on a copper substrate. A poly(methyl methacrylate) (PMMA) layer was spin coated onto the surface of graphene at 1700 rpm for 45 seconds. After curing the polymer, the copper was etched using 0.3 M ammonium persulfate to leave the graphene free and held by the PMMA. Copper etching was conducted by starting with an ammonium persulfate solution at 40 °C which was slowly brought to room temperature for 2 hours. After the copper was completely removed, the graphene was cleaned in a water bath and then transferred directly onto the surface of the device, centered on the source and drain metal contacts. The devices were left in air to dry for 5 – 6 hours, and then placed in a vacuum overnight to completely remove any moisture. Finally, PMMA was removed using an acetone bath, and the graphene channel was shaped again using S1813 positive photoresist and photolithography, after

which the errant graphene was removed using pure oxygen etching at 30 W for 4 minutes. Finally, a protective dielectric layer was deposited on the surface of the graphene channel and the devices were fully functional.[1-6] A schematic diagram showing the fabrication steps of the Gr-FET photodetector is illustrated in Figure S1.

2. Raman spectrum

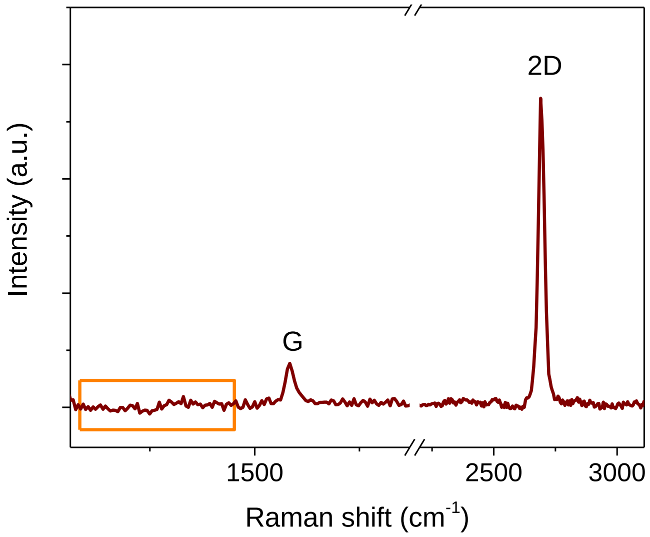

Figure S2. The Raman spectrum of a single-layer graphene after transferring on a SiO_2/Si wafer.

The quality of the single-layer graphene on a SiO_2/Si substrate was confirmed by Raman spectroscopy. Measurements were performed using a micro-Raman spectrometer (WITec UHTS 300) under laser illumination with a wavelength of 663.1 nm. Two main Raman peaks are observed at ~ 2690 cm^{-1} (2D band) and ~ 1585 cm^{-1} (G band), as shown in Figure S2. The 2D band has an FWHM of 32 cm^{-1}, containing only a single component. The ratio of the signal intensities of the 2D band to the G band, I_{2D}/I_G, is 6.5, confirming the high quality of the single-layer graphene.[7] Furthermore, defects from graphene due to the transfer process of graphene were not observed in the Raman spectrum at ~ 1350 cm^{-1} (D band).[8] High-quality of the single-layer graphene is obtained after the transfer process.

3. Optical and electrical setups

The characteristics of these Gr-FET photodetectors with and without illumination can be characterized by employing electronic systems to measure the current between the source and drain under various conditions. Keithley 2450 units were used to apply a voltage between the source and drain contacts, as well as to control the back gate bias voltage. In addition, the power source that sets the source-drain voltage also collects the drain current, characterizing the photocurrent of the device. The

back gate voltage was varied from -25 V to 25 V to obtain the I-V measurements. A lock-in amplifier was used to collect the photocurrent under low illumination conditions.

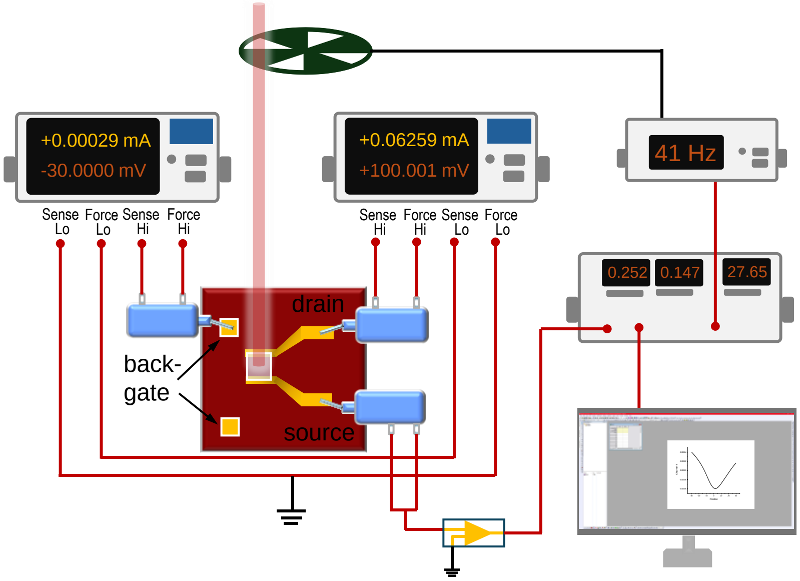

Figure S3. Electrical setup has been used to collect photocurrent. The setup includes source-meter units (Keithley 2450), a lock-in amplifier, and an oscilloscope.

Several light sources were used to illuminate the devices under the testing conditions, including laser sources with wavelengths of 532 and 1530 nm. In addition, a tungsten lamp was used in conjunction with bandpass filters for wavelengths between the visible and near-IR regions. These light sources are modulated to a specific frequency, either by using a frequency chopper wheel or a mechanical shutter placed in the path of the beam. To control the power of the light source, a half-wave plate and polarizing beam-splitter, along with neutral density filters, were used. A beam expander was employed to control the beam size, and the devices were placed in a black-anodized chamber to prevent ambient light from contributing to the measurement.

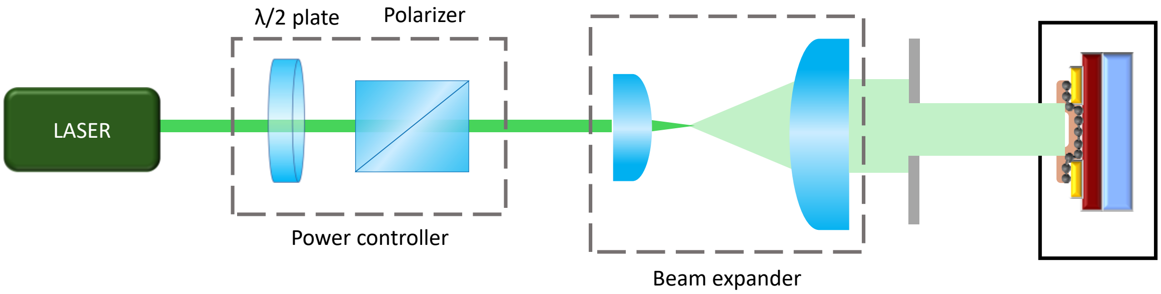

Figure S4. Optical set-up has been used to characterize photo-response of GFET photodetectors. Devices were put in a black anodized aluminum chamber to prevent random light from ambient.

4. Some current − voltage transfer curves

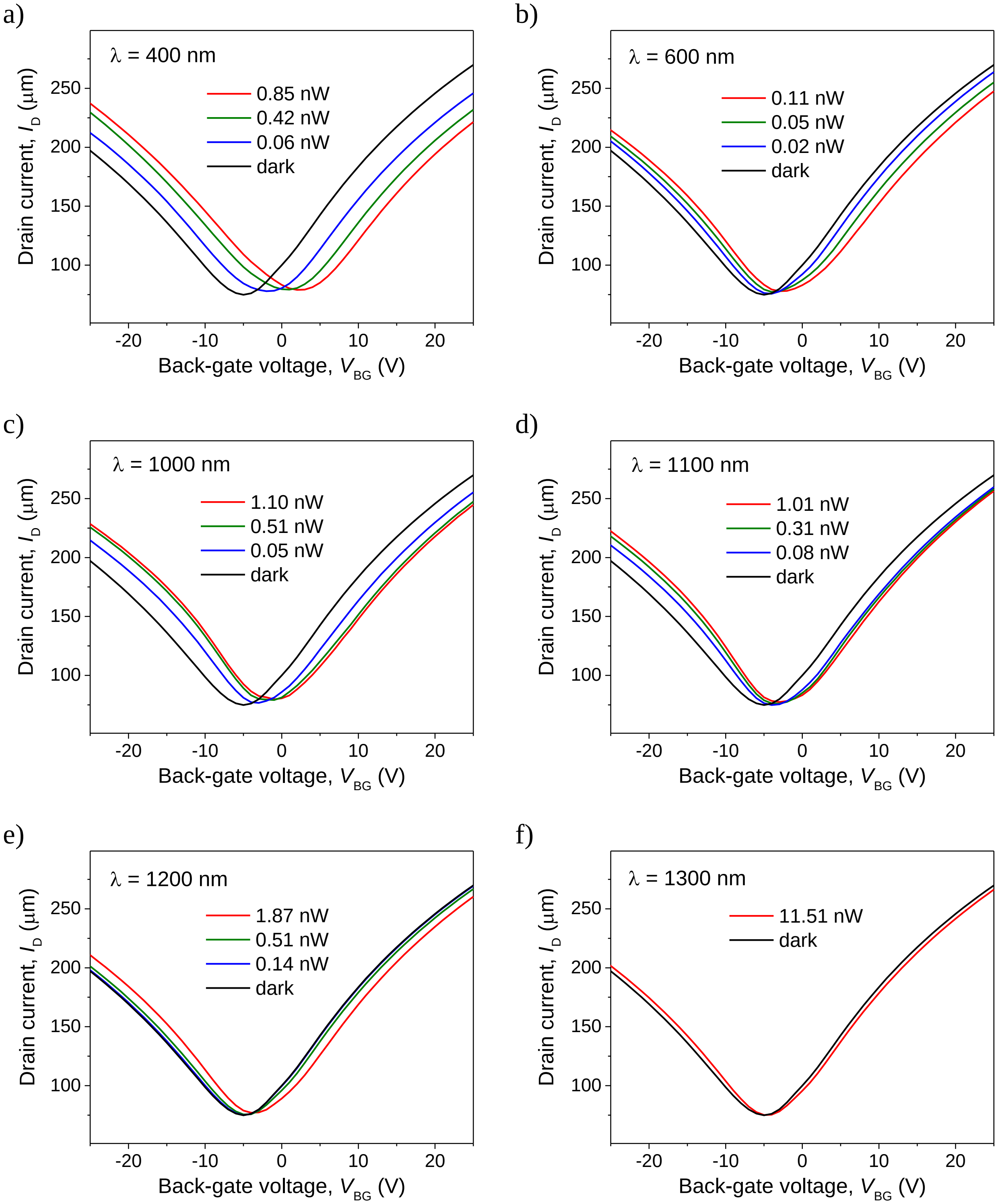

Figure S5. Current − voltage transfer curves of the GFET photodetector with and without illumination at some illumination wavelengths, including a) 400 nm, b) 600 nm, c) 1000 nm, d) 1100 nm, e) 1200, and f) 1300 nm.

5. Extra simulation results

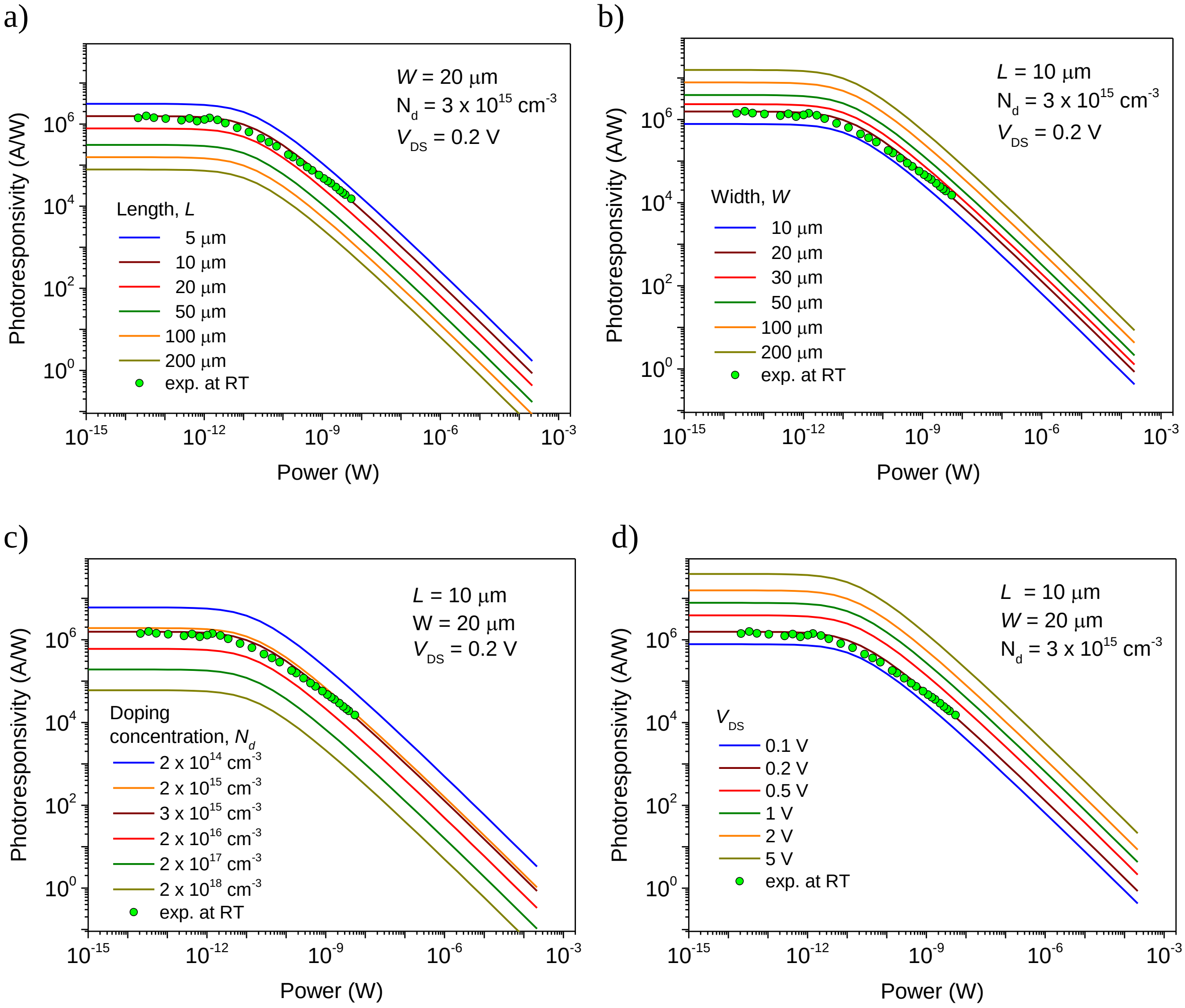

Figure S6. Computational simulations for the photoresponsivity of the GFET to predict optical performance with different structure parameters, including a) the length, *L*, b) the width, *W*, of the graphene channel, c) doping concentration, N_d, of the p-Si, and d) the voltage, V_{DS}, applied on the device.

References

(1) Ho, V. X.; Wang, Y.; Cooney, M. P.; Vinh, N. Q. Graphene-Ta_2O_5 Heterostructure Enabled High Performance, Deep-Ultraviolet to Mid-Infrared Photodetection. *Nanoscale* **2021,** *13*, 10526-10535.

(2) Wang, Y.; Ho, V. X.; Henschel, Z. N.; Cooney, M. P.; Vinh, N. Q. Effect of High-K Dielectric Layer on 1/f Noise Behavior in Graphene Field-Effect Transistors. *ACS Appl. Nano Mater.* **2021,** *4*, 3647-3653.

(3) Wang, Y. F.; Ho, V. X.; Pradhan, P.; Cooney, M. P.; Vinh, N. Q. Interfacial Photogating Effect for Hybrid Graphene-Based Photodetectors. *ACS Appl. Nano Mater.* **2021,** *4*, 8539-8545.

(4) Ho, V. X.; Wang, Y.; Cooney, M. P.; Vinh, N. Q. Graphene-Based Photodetector at Room Temperature. *Proc. SPIE* **2018,** *10729*, 1072907.

(5) Wang, Y.; Ho, V. X.; Pradhan, P.; Cooney, M. P.; Vinh, N. Q. Graphene-Germanium Quantum Dot Photodetector with High Sensitivity. *Proc. SPIE* **2019,** *11088*, 1108809.

(6) Wang, Y.; Ho, V. X.; Henschel, Z. N.; Pradhan, P.; Howe, L.; Cooney, M. P.; Vinh, N. Q. Graphene Photodetector Based on Interfacial Photogating Effect with High Sensitivity. *Proc. SPIE* **2020,** *11503*, 1150306.

(7) Malard, L. M.; Pimenta, M. A.; Dresselhaus, G.; Dresselhaus, M. S. Raman spectroscopy in graphene. *Phys. Rep.* **2009,** *473*, 51-87.

(8) Her, M.; Beams, R.; Novotny, L. Graphene transfer with reduced residue. *Phys. Lett. A* **2013,** *377*, 1455-1458.
